# Perinatal health outcomes of East African immigrant populations in Victoria, Australia: a population based study

**DOI:** 10.1186/s12884-016-0886-z

**Published:** 2016-04-26

**Authors:** Fetene B. Belihu, Mary-Ann Davey, Rhonda Small

**Affiliations:** Judith Lumley Centre, La Trobe University, 215 Franklin Street, Melbourne, VIC 3000 Australia; Department of Obstetrics and Gynaecology, Monash University, 246 Clayton Road, Clayton, VIC 3168 Australia

**Keywords:** Perinatal health, East Africa, Eritrea, Ethiopia, Somalia, Sudan, Victoria, Australia

## Abstract

**Background:**

Sub-Saharan African women are often treated as a single group in epidemiological studies of immigrant birth outcomes, potentially masking variations across countries.

**Methods:**

Cross-sectional population-based study of 432,567 singleton births in Victoria, Australia comparing mothers born in one of four East African countries (453 Eritreans, 1094 Ethiopians, 1,861 Somali and 1,404 Sudanese) relative to 427,755 Australian-born women was conducted using the Victorian Perinatal Data Collection. Pearson’s chi-square test and logistic regression analyses were performed to investigate disparities and estimate risks of perinatal mortality and other adverse perinatal outcomes after adjustment for confounders selected a priori.

**Results:**

Compared with mothers born in Australia, East African immigrants as a group had elevated odds of perinatal mortality (OR_adj_1.83, 95 % CI 1.47, 2.28), small for gestational age births (SGA) (OR_adj_1.59 95 % CI 1.46, 1.74), very low birthweight (OR_adj_1.33, 95 % CI 1.11, 1.58) and very preterm birth (OR_adj_1.55, 95 % CI 1.27, 1.90). However, they had lower odds of preterm birth (OR_adj_0.86 95 % CI 0.76, 0.98) and macrosomia (OR_adj_0.65 95 % CI 0.51, 0.83). Individual country of birth analyses indicated significant variations, with Eritrean women having higher odds of very low birthweight (OR_adj_1.80, 95 % CI 1.09, 2.98), very preterm birth (OR_adj_ 1.96, 95 % CI 1.08, 3.58), small for gestational age births (OR_adj_ 1.52, 95 % CI 1.14, 2.03) and perinatal mortality (OR_adj_ 2.69, 95 % CI 1.47, 4.91). Sudanese women had higher odds of low birthweight (OR_adj_ 1.36, 95 % CI 1.10, 1.68), very low birthweight (OR_adj_ 1.53, 95 % CI 1.13, 2.07), very preterm birth (OR_adj_ 1.78, 95 % CI 1.26, 2.53), small for gestational age births (OR_adj_ 2.13, 95 % CI 1.84, 2.47) and perinatal mortality (OR_adj_ 2.10, 95 % CI 1.44, 3.07)]. Ethiopian women differed from Australian-born women only in relation to higher odds of very preterm birth, (OR_adj_1.70 95 % CI 1.16, 2.50), and only Somali-women had significantly lower odds of preterm birth (OR_adj_0.70 95 % CI 0.56, 0.88).

**Conclusions:**

Overall, East African countries of birth were associated with increased perinatal death and some adverse perinatal outcomes; suggesting the need for strategies to enhance surveillance and health care delivery for these women. Analysis by individual country of birth groups has shown women from Eritrea and Sudan are particularly at increased risk of adverse outcomes, demonstrating the importance of antenatal identification of maternal country of birth.

**Electronic supplementary material:**

The online version of this article (doi:10.1186/s12884-016-0886-z) contains supplementary material, which is available to authorized users.

## Background

Unfavourable birth outcomes such as preterm birth, low birthweight, macrosomia, small for gestational age birth and lower Apgar score are strongly associated with neonatal morbidity, mortality and perinatal injuries [1–3]. Unfavourable birth outcomes are also known to influence early childhood and adult health; particularly, childhood behavioural problems, school performance, cardiovascular diseases and increased risk of disability pension dependency [4–6].

However, these adverse birth outcomes do not affect all populations equally, and disparities in adverse birth outcomes by ethnicity have been previously documented [7]. Immigrants’ and host country populations’ birth outcomes are also known to vary [8–12], with the magnitude and direction of variations dependent on the birth outcome studied [13], the origin and destination of immigrants [12], duration of residence in the host country [11], and migration streams [14]. Mostly, refugee and humanitarian entrants in developed countries are at higher risk of unfavourable outcomes [14, 15]. The possible reasons for disparities in adverse birth outcomes between immigrants and receiving country populations include the migration selection process, baseline health differences, maternity health care access and utilization, unfamiliarity with host country health care systems, host country language proficiency, cultural factors such as family support systems, socio-economic and genetic factors [16]. Despite immigrants often coming from poorer socio-economic contexts and socio-economic disadvantages post-migration, there is also evidence of better or equal neonatal outcomes for some immigrants [17].

Australia is one of the world’s most multicultural countries: built on migration, but with changing migration patterns. Unlike earlier times, the vast majority of immigrants entering Australia are now from non-European countries [18]. Particularly, immigrants from Africa and Asia are increasing. The country also accepts significant numbers of refugees for permanent resettlement under a humanitarian program, about one-third of these settling in the State of Victoria each year [19, 20]. In the 1990s the focus of the Australian humanitarian program shifted to Africa, the Middle East, and South West Asia [18]. Humanitarian arrivals substantially increased from African countries, particularly from Eritrea, Ethiopia, Somalia and Sudan during this period [18].

More than one in four women giving birth in Australia is foreign born [21]. African and Asian born women make up about 2 % and 15 % of births, nationally [21]. Even so, there is a scarcity of evidence from Australia about the pregnancy outcomes of African communities in general, and East African immigrants in particular. Similarly, only a few studies have reported on pregnancy outcomes of specific East African immigrant populations internationally. Three describe birth outcomes for Ethiopian-born women [10, 22, 23], three are of Somali-born women [8, 24, 25], and one report [26] focuses on outcomes for women from three of the Horn of Africa countries. The aim of this study was to investigate perinatal health outcomes of women from four east African countries (Eritrea, Ethiopia, Somalia and Sudan). These groups were chosen for their similar recent migration histories, where most have arrived via humanitarian and refugee programs, with potentially elevated risk of adverse perinatal outcomes. They also have some similarities in cultural practices and share borders, (with cross-border movements). The Sudanese group in particular are among the fastest growing humanitarian entrants in Australia, and are in the top ten non-English speaking maternal countries of birth among women giving birth in the state of Victoria, the perinatal outcomes of these four groups have yet to be described in Australia [27], with reports of immigrant birth outcomes often based on geographic region of origin masking country specific variations.

Therefore, we investigated disparities and estimated risks of: low birthweight, small for gestational age, preterm birth, five minute Apgar score, macrosomia and perinatal mortality for singleton births to immigrant women from Eritrea, Ethiopia, Somalia and Sudan, using a state-wide population based perinatal data collection in Victoria, Australia.

## Methods

### Study design, data source and study population

**Fig. 1:**
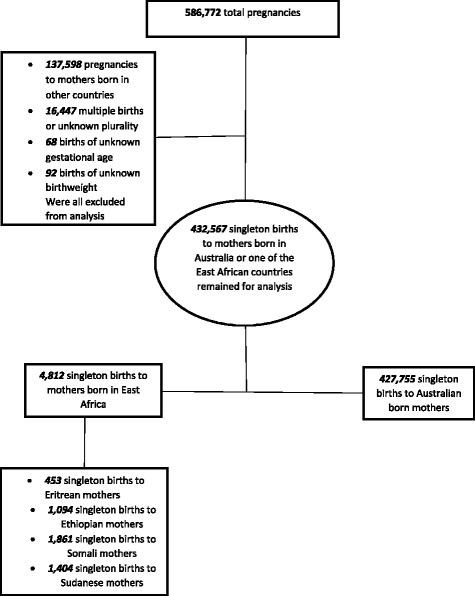
Diagrammatic description of the study population included

This is a population-based observational study of all singleton births to Australian-born and East African immigrant women using routine perinatal data in the state of Victoria, between 1999 and 2007. We accessed data from the Victorian Perinatal Data Collection (VPDC). The VPDC was established in 1982 under Victoria’s public health legislation as a population based surveillance system to collect and analyse information in relation to the health of mothers and babies to contribute to improvements in their health. Data are checked for completeness on submission to the VPDC and inconsistencies or incomplete data are queried with the reporting hospital. Internal validation of the quality and reliability of data is regularly performed to verify accuracy. A previous validity study indicated high accuracy for variable items reported to the VPDC (e.g., birthweight 99 %, estimated gestational age 92 % and maternal country of birth 93 %) [28]. Victoria is the second most populous state in Australia, known for its multicultural population [19]. All births at or after 20 weeks of gestation, or birthweight of 400gm or more if of unknown gestational age, are reported to the VPDC by hospitals, birth centres and homebirth practitioners. Data include information on maternal characteristics (including self-reported maternal country of birth), medical and obstetric conditions, procedures in labour and birth, and maternal and infant outcomes. The study population comprised all singleton births to mothers born in Australia or one of the East African countries (Eritrea, Ethiopia, Somalia and Sudan). These four East African countries were purposely selected as they have similar migration histories, mainly arriving in Australia via humanitarian and refugee programs, and are therefore likely to be at elevated risk of adverse perinatal outcomes post-migration. As shown in Fig. 1, there were 586,772 pregnancies in Victoria, Australia between 1999 and 2007. Of these, 137,598 births to mothers born in countries other than Australia or one of the East African countries, 16,447 multiple births or births of unknown plurality, 92 births of missing birthweight and 68 births of unknown gestational age were excluded. Therefore, 432,567 singleton births remained for analysis. There was no difference between East African immigrants and Australian-born women in births with missing birthweight or gestational age.

### Outcome measures

Our outcomes of interest were low and very low birthweight, preterm and very preterm birth, small for gestational age (SGA), five minute Apgar score <7(for live born infants at 37 or more weeks gestation), macrosomia, and perinatal mortality (stillbirth and neonatal death). All outcome variables were categorised to generate a binary variable to fit binary logistic regression models for the analyses, and coded as “yes” if the outcome of interest was present and “no” if absent (eg. very low birthweight is yes if birthweight is <1,500 g and no if birthweight > =1500gm).

Low and very low birthweight were defined as weights <2,500 and <1,500 grams at birth respectively. Birthweight data are based on the first weight of both stillborn and live-born babies usually taken within the first hour postpartum, irrespective of gestational age. Macrosomia was defined as birthweight of 4,500 grams or more. Preterm and very preterm births were defined as births before 37 and 32 completed weeks of gestation. SGA included gestational age and sex specific birthweight below the 10^th^ centile for gestational age based on the Australian national birthweight chart [29]. Gestational age was estimated from the first date of the last ‘normal’ menstrual period and/or early obstetric ultrasound usually before 12 weeks of gestation. Stillbirths (fetal death) were those deaths before the complete expulsion or extraction of a product of conception at 20 or more weeks of gestation or 400 grams or more, if gestational age is unknown. Neonatal deaths are those deaths within 28 days of birth among the live-born. Therefore, perinatal mortality includes stillbirths and neonatal death up to 28 days postpartum.

### Exposure variables

Our main exposure variable, maternal country of birth is one of the routine items reported in the perinatal data. All women born in Australia, Eritrea, Ethiopia, Somalia or Sudan were included. A single East African group was also created by pooling all four immigrant source country groups. Potential confounders considered were maternal age, marital status, parity, maternal medical and obstetric conditions, socio-economic index for areas (a measure of relative area specific advantage and disadvantage based on postcodes of residence) and year of birth.

Maternal age at the time of birth was divided in to three groups: those under 20 years, 20–34 years and those 35 and over. In all multivariable binary logistic regression analyses, mothers within the age group 20–34 were used as a reference. Marital status at the time of birth was classified into three groups: married, defacto and single (including, widowed, separated and divorced). Married women were used as a reference category in all our multivariable analyses. Admission status of mothers was whether the mothers were admitted as a public or private patient during the index child birth. Parity was classified as primiparous, for a first birth and, multiparous for second or subsequent births. Multiparous women were the reference category in all multivariable analyses involving parity. Maternal medical conditions refer to presence of any pre-existing medical condition that affected the index pregnancy or its management (e.g., pre-existing essential hypertension, diabetes mellitus, renal disease, mental disorder and diseases of the nervous system, other cardiovascular diseases) and coded as yes/no.

Income and education are not routinely collected in the VPDC, but postcode of residence is, so women’s socio-economic status was measured using the 2006 Australian Bureau of Statistics’ (ABS) Index of Relative Socio-economic Advantage and Disadvantage (IRSAD) [30], which uses aggregated statistical local area household characteristics to measure relative area specific advantage and disadvantage [30]. Each birth record was given an IRSAD index score using VPDC place of residence postcode data, then re-scored to an ordinal ranking. These IRSAD rankings were then grouped (using sample tertiles) as living in either ‘low’, ‘medium’ or ‘high’ socio-economic areas and reported in descriptive analyses. In multivariable analyses the continuous version of IRSAD scores was directly entered into the model.

### Statistical analysis

We used Stata version 11.0 to analyse the data (Stata Corp LP, College Station, TX). In descriptive analyses we examined frequencies and percentages of adverse perinatal health outcomes and perinatal mortality by maternal country of birth. Pearson’s chi-square test was used to test for differences in adverse perinatal outcomes between infants of each East African maternal country of birth and Australian-born mothers. For each outcome studied, we calculated crude and adjusted odds ratios with 95 % confidence intervals to examine the association of maternal country of birth with adverse perinatal outcomes and perinatal death. An adjusted regression model with 95 % confidence interval is presented for each outcome, adjusting for maternal age, parity, marital status, IRSAD, year of birth and for the occurrence of any maternal medical condition that affected the index pregnancy or its management. These confounders were selected a priori based on previous literature reporting on their potential associations with birth outcomes and migration [31]. Year of the index birth was included as a covariate to control for secular trends in adverse outcomes. Missing data were dealt with as separate subcategories within each variable. A sensitivity analysis was conducted to assess the impact of limiting analyses to births at 22 or more weeks or birthweight of 500gm, (a commonly agreed viability threshold). Results are presented for each East African country and for the pooled East African group. All comparisons are with Australian-born mothers.

## Results

Tables 1 and 2 show the characteristics and selected perinatal health outcomes of East African immigrants and Australian-born women. East African countries contributed 1.1 % of singletons studied. However, births to East African immigrant groups saw a substantial increase over time, tripling from 0.6 % between 1999 and 2001 to 1.7 % between 2005 and 2007.

**Table 1:** Characteristics of Australian and East African- born women giving birth to singletons between 1999 and 2007 in Victoria, Australia

	Australia	Eritrea		Ethiopia		Somalia		Sudan		Total East Africa	
Factor		n (%)	*P*-values	n (%)	*P*-values	n (%)	*P*-values	n (%)	*P*-values	n (%)	*P*-values
Obs (%)	427,755 (98.9)	453 (0.10)		1094 (0.25)		1861 (0.43)		1404 (0.32)		4,812 (1.1)	
Age (years)											
<20	14,688 (3.4)	<5		21 (1.9)		51 (2.7)		87 (6.2)		161 (3.3)	
20-34	325,885 (76.2)	360 (79.5)	0.002	858 (78.5)	0.016	1483 (79.7)	0.002	1131 (80.6)	0.001	3832 (79.6)	0.001
35+	87,154 (20.4)	91 (20.1)		215 (19.6)		327 (17.6)		186 (13.2)		819 (17.1)	
Missing	28 (0.01)	0(0)		0 (0)		0 (0)		0 (0)		0 (0)	
Marital status											
Single	59,747 (14.0)	71 (15.7)		313 (28.7)		356 (19.1)		195 (13.9)		935 (19.4)	
Married	299,104 (69.9)	368 (81.2)	<0.001	727 (66.4)	<0.001	1,472 (79.1)	<0.001	1,172 (83.5)	<0.001	3,739 (77.7)	0.001
Defacto	68,413 (16)	12 (2.7)		51 (4.7)		28 (1.5)		28 (2.0)		119 (2.5)	
Missing	491 (0.1)	2 (0.4)		3 (0.3)		5 (0.3)		9 (0.6)		19 (0.4)	
Parity											
Primipara	182,381 (42.6)	171 (37.8)		384 (35.2)		378 (20.3)		360 (25.6)		1,293 (26.9)	
Multipara	245,372 (57.4)	282 (62.2)	0.035	710 (64.8)	<0.001	1,483 (79.7)	<0.001	1,044 (74.4)	<0.001	3,519 (73.1)	0.001
Missing	2(0)	0 (0)		0 (0)		0 (0)		0 (0)		0 (0)	
Hospital admission status											
Public	267,023 (62.4)	443 (97.8)		1,059 (96.7)		1,845 (99.1)		1,364 (97.2)		4,711 (97.9)	
Private	160,732 (37.6)	10 (2.2)	<0.001	35 (3.3)	<0.001	16 (0.9)	<0.001	40 (2.8)	<0.001	101 (2.1)	<0.001
^a^IRSAD scores											
High	118,771 (27.8)	103 (22.7)		117 (10.8)		348 (18.7)		148 (10.5)		716 (15.0)	
Medium	166,849 (39.0)	246 (54.3)	<0.001	588 (53.7)	<0.001	861 (46.3)	<0.001	377 (26.8)	<0.001	2,072 (43.0)	<0.001
Low	140,442 (32.8)	96 (21.2)		388 (35.4)		651 (35)		878 (62.6)		2,013 (41.8)	
Missing	1,693 (0.4)	8 (1.8)		1 (0.1)		1 (0.1)		1 (0.1)		11 (0.2)	
Year of birth											
1999-2001	137,801 (32.2)	110 (24.3)		157 (14.3)		501 (26.9)		78 (5.6)		846 (17.6)	
2002-2004	139,776 (32.7)	159 (35.1)	0.001	354 (32.3)	<0.001	609 (32.7)	<0.001	298 (21.2)	<0.001	1,420 (29.5)	<0.001
2005-2007	150,178 (35.1)	184 (40.6)		583 (53.3)		751 (40.4)		1,028 (73.2)		2,546 (52.9)	
Any maternal medical complication											
Yes	112,798 (26.4)	146 (32.2)		336 (30.8)		615 (33.1)		486 (34.6)		1,583 (32.9)	
No	314,957 (73.6)	307 (67.8)	0.005	758 (69.2)	0.001	1,246 (66.9)	<0.001	918 (65.4)	0.001	3,229 (67.1)	<0.001

^*a*^ *IRSAD* index of relative socio-economic advantage and disadvantage, *p*-values are the results of chi-square test comparing each East African countries with Australian-born women

**Table 2:** Selected perinatal health outcomes of Australian and East African-born women giving birth to singletons in Victoria, Australia between 1999 and 2007

Country of birth	Australia (*N* = 427,755)	Eritrea (*N* = 453)		Ethiopia (*N* = 1,094)		Somalia (*N* = 1,861)		Sudan (*N* = 1,404)		Total East Africa (*N* = 4,812)	
Outcome	n (%)	n (%)	*P*-values	n (%)	*P*-values	n (%)	*P*-values	n (%)	*P*-values	n (%)	*P*-values
Very low birthweight	9,149 (2.1)	16 (3.5)	0.046	36 (3.3)	0.006	39(2.1)	0.837	44 (3.1)	0.013	135 (2.8)	0.002
Low birthweight	22,502 (5.3)	30 (6.6)	0.197	57 (5.2)	0.964	84(4.5)	0.147	94 (6.7)	0.017	265 (5.5)	0.424
Macrosomia	8,564 (2.0)	6 (1.3)	0.348	15 (1.4)	0.177	37(2.0)	0.859	10 (0.7)	0.001	68 (1.4)	0.005
Preterm birth	26,900 (6.3)	28 (6.2)	0.916	70 (6.4)	0.895	82(4.4)	0.001	83 (5.9)	0.549	263 (5.5)	0.018
Very preterm birth	5,680 (1.3)	11 (2.4)	0.049	28 (2.6)	0.001	28(1.5)	0.581	33 (2.4)	0.001	100 (2.1)	0.001
5’ Apgar Score <7^a^	3,776 (0.94)	6 (1.4)	0.165	7 (0.68)	0.736	16(0.90)	0.744	18 (1.4)	0.106	47 (1.0)	0.508
Small for gestational age	37,820 (8.8)	54 (11.9)	0.021	104 (9.5)	0.443	222 (11.9)	0.001	219 (15.5)	0.001	599 (12.4)	0.001
Perinatal mortality (n/1000 births)	4,205 (9.8)	11 (24.3)	0.002	16(14.6)	0.069	31(17)	0.004	28(20)	0.001	86 (18)	0.001

*P*-values are the Pearson’s chi-square test result comparing each maternal country of birth with Australian-born mothers

^a^Computed for term live born infants

Maternal age, marital status, parity, hospital admission status, IRSAD groups, and maternal medical conditions all varied considerably between Australian-born and East African-born mothers (Table 1). East African-born immigrants were more likely to be multiparous, to live in the most socio-economically deprived areas, to be married, to be admitted as public patients and to suffer from one or more medical complications. However, they were less likely to be over 35 years of age. Variations in age and IRSAD within immigrant countries of birth were also apparent, with Sudanese immigrants in particular much less likely to be older than 35 years and more likely to live in a deprived area.

Table 2 presents the findings of chi-square tests of association which demonstrate that East African immigrants as a group had significantly higher rates of very low birthweight, very preterm births, perinatal mortality and small for gestational age births. However, the findings differed for individual countries. Perinatal mortality was not significantly higher for women born in Ethiopia. Eritrean and Sudanese immigrants particularly suffered from very high perinatal mortality, 24 and 20 per 1000 births respectively. The corresponding figure for the Australian-born population was 9.8 per 1000 births. The square values based on univariable analyses however indicated lower rates of preterm birth and macrosomia for the combined East African group. Macrosomia was particularly lower for Sudanese women, while preterm birth was significantly lower only for Somali women.

Table 3 shows the results of crude and adjusted binary logistic regression analyses. Maternal country of birth remained a significant risk factor for most outcomes, even after accounting for maternal demographic, socio-economic and medical risk factors.

**Table 3:** Crude and adjusted odds ratios with (95%CI) for selected perinatal health outcomes of East African immigrants and Australian-born women giving birth to singletons in Victoria, Australia between 1999 and 2007

Outcomes	Australia	Eritrea	Ethiopia	Somalia	Sudan	Total East Africa
Low birthweight (n and %)	22,502 (5.3)	30 (6.6)	57 (5.2)	84 (4.5)	94 (6.7)	265 (5.5)
OR (95 % CI)	1.00	1.28 (0.88, 1.85)	0.99(0.76, 1.29)	0.85 (0.68, 1.06)	**1.29 (1.05, 1.59)**	1.05 (0.93, 1.19)
OR_adj_ (95 % CI)	1.00	1.39 (0.95, 2.02)	0.90 (0.68, 1.17)	0.89 (0.71, 1.11)	**1.36 (1.10, 1.68)**	1.07 (0.94, 1.21)
Very low birthweight (n and %)	9,149 (2.2)	16 (3.5)	36 (3.3)	39 (2.1)	44 (3.1)	135 (2.8)
OR (95 % CI)	1.00	**1.68 (1.02, 2.76)**	**1.56 (1.12, 2.17)**	0.98 (0.71, 1.35)	**1.48 (1.08, 2.00)**	**1.32 (1.11, 1.57)**
OR_adj_ (95 % CI)	1.00	**1.80 (1.09, 2.98)**	1.39 (0.99, 1.96)	1.01 (0.73, 1.40)	**1.53 (1.13, 2.07)**	**1.33 (1.11, 1.58)**
Preterm birth (n and %)	26,900 (6.3)	28 (6.2)	70 (6.4)	82 (4.4)	83 (5.9)	263 (5.5)
OR (95 % CI)	1.00	0.98 (0.67,1.44)	1.02 (0.80, 1.30)	**0.69 (0.55, 0.86)**	0.94 (0.75,1.17)	**0.86 (0.76, 0.98)**
OR_adj_ (95 % CI)	1.00	1.02 (0.70, 1.50)	0.95 (0.74, 1.21)	**0.70 (0.56, 0.88)**	0.96 (0 .76, 1.19)	**0.86 (0.76, 0.98)**
Very preterm birth (n and %)	5,680 (1.4)	11 (2.4)	28 (2.6)	28 (1.5)	33 (2.4)	100 (2.1)
OR (95 % CI)	1.00	**1.85 (1.02, 3.37)**	**1.95 (1.34, 2.84)**	1.14 (0.78, 1.65)	**1.79 (1.27, 2.53)**	**1.58 (1.29, 1.93)**
OR_adj_ (95 % CI)	1.00	**1.96 (1.08, 3.58)**	**1.70 (1.16, 2.50)**	1.18 (0.81, 1.71)	**1.78 (1.26, 2.53)**	**1.55 (1.27, 1.90)**
Apgar score <7 (n and %)	3,776 (0.94)	6 (1.4)	7(0.68)	16 (0.90)	18 (1.4)	47 (1.0)
OR (95 % CI)	1.00	1.50 (0.67, 3.37)	0.72 (0.34, 1.52)	0.96 (0.59, 1.57)	1.47 (0.92, 2.34)	1.10 (0.83, 1.47)
OR_adj_ (95 % CI)	1.00	1.62 (0.72, 3.64)	0.72 (0.35, 1.53)	1.08 (0.66, 1.78)	1.56 (0.97, 2.49)	1.18 (0.89, 1.59)
Macrosomia (n and %)	8,564 (2)	6 (1.3)	15 (1.4)	37 (2)	10 (0.7)	68 (1.4)
OR (95 % CI)	1.00	0.66 (0.29, 1.47)	0 .68 (0.41, 1.13)	0.99 (0.72, 1.38)	**0.35 (0.19, 0.65)**	**0.70 (0.55, 0.89)**
OR_adj_ (95 % CI)	1.00	0 .65 (0.29, 1.45)	0.67 (0.40, 1.11)	0.90 (0.65, 1.25)	**0.32 (0.17, 0.59)**	**0.65 (0.51, 0.83)**
SGA (n and %)	37,820 (8.8)	54 (11.9)	104 (9.5)	222 (11.9)	219 (15.5)	599 (12.4)
OR (95 % CI)	1.00	**1.40 (1.05, 1.85)**	1.08 (0.88, 1.33)	**1.40 (1.21, 1.61)**	**1.91 (1.65, 2.20)**	**1.47 (1.34, 1.60)**
OR_adj_ (95 % CI)	1.00	**1.52 (1.14, 2.03)**	1.07 (0 .87, 1.31)	**1.58 (1.37, 1.83)**	**2.13 (1.84, 2.47)**	**1.59 (1.46, 1.74)**
Perinatal mortality (n/1000)	4,205 (9.8)	11 (24)	16 (14.6)	31 (17)	28 (20)	86 (18)
OR (95 % CI)	1.00	**2.51 (1.38, 4.56)**	1.49 (0.91, 2.45)	**1.71 (1.19, 2.44)**	**2.05 (1.41, 2.98)**	**1.83 (1.48, 2.27)**
OR_adj_ (95 % CI)	1.00	**2.69 (1.47, 4.91)**	1.29 (0.77, 2.15)	**1.78 (1.25, 2.55)**	**2.10(1.44, 3.07)**	**1.83 (1.47, 2.28)**

*OR* crude odds ratio

*OR*_*adj*_ adjusted for maternal age, parity, marital status, SEIFA, year of birth and maternal medical complications, Bold indicate statistically significant differences

In unadjusted and adjusted regression analysis, East African immigrants as a group had significantly higher odds of very low birthweight, very preterm birth, SGA births, and perinatal mortality. But they had lower odds of preterm birth and macrosomia. However, individual East African countries had varied outcomes after accounting for a wide range of confounders. Only Somali born women had significantly lower odds of preterm births. Sudanese-born mothers alone had significantly lower odds of macrosomia and higher odds of low birthweight. SGA births and perinatal mortality were significantly elevated for all East African countries after adjustment, except Ethiopia. Disaggregated analysis (not shown by individual maternal countries birth due to small numbers in some groups), indicated that the observed increased odds of perinatal mortality for immigrants from East Africa was mainly due to fetal death. Overall, 66 out of 86 (77 %) perinatal deaths were due to stillbirth. A sensitivity analysis conducted to assess the impact of limiting analyses to births at 22 or more weeks or birthweight of 500gm, (a commonly agreed viability threshold) did not alter our findings (Additional file 1).

## Discussion

### Main findings

Our investigation suggests considerable variation in perinatal health outcomes between women born in Australia and those born in East Africa, and among the four East African immigrant groups studied. As a group combined, and after adjustment for potential confounders, East African women experienced elevated odds of SGA, very low birthweight, very preterm birth and perinatal mortality, but lower odds of preterm birth and macrosomia. The combined East African immigrant group suffered from an 83 % increase in the odds of perinatal mortality, but this excess mortality varied across countries, with women from Sudan and Eritrea showing the most adverse perinatal mortality rates. Similarly, East African immigrants were more likely to give birth to SGA babies but this also varied between countries, with no elevated risk found among women from Ethiopia. Although our comparisons are with Australian born mothers, it is clear that the East African countries also varied in the extent to which they differed from the Australian-born population for low birthweight, very low birthweight and very preterm birth.

### Interpretation

Our analysis has shown that risk of perinatal mortality and some adverse birth outcomes among some East African immigrants remained strong, even after adjusting for a number of potential confounders. Our finding of elevated perinatal death is consistent with findings from immigrant studies in Australia and other countries, such as Sweden [32], South Asian women in Australia [7], and Somali women in six countries [8]. Similarly, systematic reviews of immigrant birth outcome studies have indicated Sub-Saharan Africans as a group [9], and refugees [15] in general are most vulnerable for increased fetal and neonatal deaths. Increased SGA births were also observed for Somali women and East African couples in Sweden [25, 33]. Consistent with our findings, Somali women have been reported to have lower odds of preterm births in six industrialized countries [8], and East African immigrants have been shown elsewhere to have lower risk of preterm births [33]. Another recent study from New South Wales, Australia similarly documented higher SGA and stillbirths for immigrants [34], though the authors did not investigate immigrants from individual African countries. One systematic review has indicated increased odds of low birthweight for Sub-Saharan Africans in general [12], we found the higher odds to be in very low birthweight among this East African group. Infants of immigrant Somali women were previously reported to have poorer five minute Apgar scores [8, 24] though this was not replicated in our study. Surprisingly perhaps, Ethiopian immigrants in Victoria experienced elevated risks only for very preterm births, unlike Ethiopians who migrated to Israel in whom a range of adverse perinatal outcomes were observed [23]. This might be attributed to the number of background factors we were able to control for, differences in the characteristics of the Ethiopian migrant populations or to receiving country characteristics, including access to health care. Favourable pregnancy outcomes for Ethiopian immigrants have also been documented in the United States of America (USA) [22].

Most East African immigrants have entered Australia as refugees [35]. Many have experiences of trauma fleeing war-torn countries. Often, they arrive after prolonged and stressful stays in refugee camps in transit [35], and are more likely to face challenges of employment post-migration [36]. There is evidence associating maternal stress with some adverse birth outcomes, such as SGA births [37]. Moreover, the elevated SGA births among most of these immigrants may indicate a deficiency of psychosocial coping resources such as a sufficient social network, social support and control in daily life which have been found to be associated with SGA births [38]. Maternal stress may thus be implicated in the observed elevated adverse outcomes. It is also possible that lower gestational weight gain and low maternal body weight among East African immigrants could have contributed to the observed higher SGA births, although we were unable to investigate this due to lack of information on maternal weight and height in our dataset [39]. The higher rate of SGA observed among East African immigrants should be interpreted cautiously, as we used the general Australian weight chart. Lower birthweight for gestational age may reflect physiological rather than pathological differences for some. However, while customised birthweight centiles have been recommended [40], their usefulness is still debated [41].

There is a scarcity of evidence on infections such as bacterial vaginosis among immigrants to Australia, a condition previously associated with adverse birth outcomes, particularly preterm birth and low birthweight preterm births [42]. One Australian study has indicated higher rates of syphilis and hepatitis infections among African humanitarian entrants [14]. Future research may need to focus on investigating associations between infection and adverse birth outcomes among East African immigrants.

Explanations for the elevated odds of perinatal mortality in Eritrean, Somali and Sudanese women following adjustment for a wide range of confounding factors must remain speculative, but it would appear that factors beyond clinical risk may be implicated. Suboptimal perinatal care factors which could result in avoidable perinatal death have, for example, been found to be more common among East African immigrants elsewhere [26]. The elevated odds might also indicate relative social deprivation and barriers to accessing quality health care among this group of immigrants. This needs to be further clarified by future research, as our dataset did not allow us to explore the specific causes and timing of perinatal deaths.

Our findings do not however show uniformly elevated adverse perinatal health outcomes across all East African immigrant groups. Sudanese women experienced the highest number of adverse outcomes while Ethiopian-born women experienced the fewest. Accounting for this is challenging. It may be a reflection of home country maternal health status differences and varying levels of social and political upheaval, exerting influence post-migration. Sudan has for example one of the highest rates of adverse perinatal health outcomes such as SGA births world-wide [43], while more favourable outcomes for Ethiopian immigrants have also been seen in the USA [22]. Furthermore, differences in outcomes between countries may reflect differentials in levels of health care use, duration of residence and acculturation including receiving country language proficiency after migration. However, the wider 95 % confidence intervals for some outcomes (eg. perinatal mortality in Ethiopian and Eritrean-born women) might indicate that the sample size is not large enough to estimate the true odds of some of these adverse perinatal outcomes, suggesting cautious interpretation of observed differences among countries.

Given the sensitivity of perinatal mortality to quality of health care and social services [44], our finding of elevated perinatal mortality among these immigrants (particularly among Eritreans, Somali and Sudanese) raises concerns about health service delivery for East African immigrants. Mothers from East Africa might not be attending antenatal care in a timely way or not consider it relevant, hindering antenatal detection of life threatening conditions such as congenital anomalies or fetal growth restriction, with impacts on the rate of stillbirth [45]. African refugee women are likely to face complex health challenges in accessing maternity care because of their language difficulties and the possibility that their particular needs are not understood by health care staff [46]. Even if women attend antenatal care and a congenital anomaly is diagnosed, they may refuse termination of pregnancy for cultural and religious reasons [45]. Therefore, ensuring and organizing culturally and linguistically responsive health services, and engaging more actively with respective local communities could enhance participation in, and quality of pregnancy care with resultant reduced adverse perinatal outcomes among East African immigrants. Furthermore, antenatal and intrapartum audits of care among East African women might help to pinpoint causes and timing of fetal loss.

### Strengths and limitations

This study has a number of strengths. First, we have reported outcomes for specific countries of birth instead of using geographic region of birth for immigrants, which prevents masking of individual country differences in outcomes. Second, our study analysed routinely collected data on all births in Victoria and the data variables analysed have been previously validated, with high levels of accuracy [28]. Third, the significant numbers of East African immigrants included, enabled investigation of rarer outcomes such as perinatal mortality. Fourth, unlike many previous studies, we accounted for a wide range of confounders, demonstrating the independent effect of country of birth on the selected perinatal health outcomes.

The study also has some limitations. The dataset available to us for analysis was not able to be updated to include more recent data and this means that we do not know if changes have occurred in outcomes for East African women since 2007, though recent hospital-based studies would suggest that adverse outcomes remain an issue among African refugee groups in Australia [14]. It is to be hoped that the findings reported here will motivate future research and close monitoring/surveillance of birth outcomes for East African immigrant women. Important risk factors such as smoking and drug use behaviours of mothers are unknown in our study population. The prevalence of smoking and drug use among women in the immigrant source countries studied is however known to be low [47], though behavioural changes over time post-migration are possible. Level of maternity health care use during pregnancy is not available in the dataset. Although antenatal care is freely available in Australia, so may be less likely to be the explanation for our findings than in other settings, challenges in accessing quality health care services including antenatal care among African communities has been previously documented in Australia [46]. Furthermore, Humanitarian entrants in general, including those from African are known to have poor antenatal attendance and late first pregnancy care visit in Australia [48]. Therefore, we cannot rule this out as a contributing factor in the observed adverse perinatal outcomes. It was not possible to identify births to the same mother during the study period. Therefore, clustering of births is possible, but is likely to be insignificant in explaining these disparities. Other migration related factors such as refugee/non-refugee status at the individual level, English language fluency and length of residence in Australia for these immigrants were not available and hence their potential impact could not be examined. However, including duration of residence for immigrant women in routine perinatal data in the future appears appropriate, given its potential explanatory role in perinatal outcomes; a recommendation which is supported by an international Delphi study [49]. While it could be said that our study is at risk of type one error due to the large number of comparisons conducted, it should be noted that the very small *p*-values associated with our findings indicate that type one error is unlikely to be a significant issue. Finally, although we were able to control for area based socio-economic status, variations in socio-economic position at the individual level are not captured.

## Conclusion

In conclusion, in comparison with Australian-born women, East African immigrants suffered from excess perinatal mortality and a range of other adverse perinatal outcomes independently of many potential confounders. The odds of preterm birth and macrosomia were however lower. Despite seemingly similar migration histories and experiences, women from different East African countries appear to have different outcomes, demonstrating the importance and relevance of analysing and monitoring immigrant birth outcomes by individual country of birth, whenever possible. The elevated perinatal mortality in particular suggests the need for improved maternity care for East African immigrants in order to address potentially avoidable contributing factors.

## Details of ethics approval

This study was approved by La Trobe University Human Research Ethics Committee (HREC 05-062 mod 5). We also obtained approval from the Consultative Council on Obstetric and Paediatric Mortality and Morbidity to access de-identified data for this study.

## Consent to publish

Not applicable.

## Availability of data and materials

The data on which this study is based including the supplementary file (supporting the findings) are owned by the Consultative Council on Obstetric and Paediatric Mortality and Morbidity (CCOPMM) of Victoria. The data permissions which support this study do not allow for the data to be shared.

## Additional file

Additional file 1: Table S1. Crude and adjusted odds ratios with (95 % CI) for selected perinatal health outcomes of East African immigrants and Australian-born women giving birth to singletons at ≥22 weeks of gestation in Victoria, Australia between 1999 and 2007. (DOC 68 kb)

## Abbreviations

CCOPMM: consultative council on obstetric and paediatric mortality and morbidity
IRSAD: index of relative socio-economic advantage and disadvantage
LUPRS: La Trobe University Postgraduate Research Scholarship
SGA: small for gestational age
VPDC: victorian perinatal data collection

## References

[1] Casey BM, McIntire DD, Leveno KJ (2001). The continuing value of the Apgar score for the assessment of newborn infants. N Engl J Med.

[2] Lau C, Ambalavanan N, Chakraborty H, Wingate MS, Carlo WA (2013). Extremely low birth weight and infant mortality rates in the United States. Pediatrics.

[3] Meshari A, De Silva S, Rahman I (1990). Fetal macrosomia—maternal risks and fetal outcome. Int J Gynaecol Obstet.

[4] Curhan GC, Willett WC, Rimm EB, Spiegelman D, Ascherio AL, Stampfer MJ (1996). Birth weight and adult hypertension, diabetes mellitus, and obesity in US men. Circulation.

[5] Helgertz J, Vågerö D (2014). Small for gestational age and adulthood risk of disability pension: The contribution of childhood and adulthood conditions. Soc Sci Med.

[6] Moster D, Lie RT, Markestad T (2008). Long-term medical and social consequences of preterm birth. N Engl J Med.

[7] Drysdale H, Ranasinha S, Kendall A, Knight M, Wallace EM (2012). Ethnicity and the risk of late-pregnancy stillbirth. Med J Aust.

[8] Small R, Gagnon A, Gissler M, Zeitlin J, Bennis M, Glazier R (2008). Somali women and their pregnancy outcomes postmigration: data from six receiving countries. BJOG.

[9] Gagnon AJ, Zimbeck M, Zeitlin J, Collaboration R (2009). Migration to western industrialised countries and perinatal health: a systematic review. Soc Sci Med.

[10] Salim R, Mfra A, Garmi G, Shalev E (2012). Comparison of intrapartum outcome among immigrant women from Ethiopia and the general obstetric population in Israel. Int J Gynaecol Obstet.

[11] Urquia ML, Frank JW, Moineddin R, Glazier RH (2010). Immigrants’ duration of residence and adverse birth outcomes: a population-based study. BJOG.

[12] Urquia ML, Glazier RH, Blondel B, Zeitlin J, Gissler M, Macfarlane A (2010). International migration and adverse birth outcomes: role of ethnicity, region of origin and destination. J Epidemiol Community Health.

[13] Urquia ML, O'Campo PJ, Heaman MI (2012). Revisiting the immigrant paradox in reproductive health: the roles of duration of residence and ethnicity. Soc Sci Med.

[14] Gibson-Helm M, Teede H, Block A, Knight M, East C, Wallace EM (2014). Maternal health and pregnancy outcomes among women of refugee background from African countries: a retrospective, observational study in Australia. BMC Pregnancy Childbirth.

[15] Gissler M, Alexander S, MacFarlane A, Small R, Stray‐Pedersen B, Zeitlin J (2009). Stillbirths and infant deaths among migrants in industrialized countries. Acta Obstet Gynecol Scand.

[16] Pennell CE, Jacobsson B, Williams SM, Buus RM, Muglia LJ, Dolan SM (2007). Genetic epidemiologic studies of preterm birth: guidelines for research. Am J Obstet Gynecol.

[17] Hessol NA, Fuentes-Afflick E (2000). The perinatal advantage of Mexican-origin Latina women. Ann Epidemiol.

[18] Australian Government Department of Immigration and Citizenship. Australia’s Humanitarian Program - Information Paper available at http://www.immi.gov.au/media/publications/pdf/hp-client-info-paper.pdf retrieved on April, 20/2015.

[19] Australian Bureau of Statistics (2012). 2012 Year Book of Australia.

[20] Paxton GA, Rice J, Davie G, Carapetis JR, Skull SA (2011). East African immigrant children in Australia have poor immunisation coverage. J Paediatr Child Health.

[21] Hilder L, Zhichao Z, Parker M, Jahan S, Chambers G (2014). Australia's mothers and babies 2012.

[22] Wasse H, Holt VL, Daling JR (1994). Pregnancy risk factors and birth outcomes in Washington State: a comparison of Ethiopian-born and US-born women. Am J Public Health.

[23] Calderon-Margalit R, Sherman D, Manor O, Kurzweil Y (2015). Adverse Perinatal Outcomes among Immigrant Women from Ethiopia in Israel. Birth.

[24] Johnson EB, Reed SD, Hitti J, Batra M (2005). Increased risk of adverse pregnancy outcome among Somali immigrants in Washington state. Am J Obstet Gynecol.

[25] Råssjö EB, Byrskog U, Samir R, Klingberg-Allvin M (2013). Somali women’s use of maternity health services and the outcome of their pregnancies: a descriptive study comparing Somali immigrants with native-born Swedish women. Sex Reprod Healthc.

[26] Essen B, Bodker B, Sjoberg NO, Langhoff-Roos J, Greisen G, Gudmundsson S (2002). Are some perinatal deaths in immigrant groups linked to suboptimal perinatal care services?. BJOG.

[27] Consultative Council on Obstetric and Paediatric Mortality and Morbidity 2014, 2010 and 2011, Victoria’s Mothers and Babies, Victoria’s Maternal, Perinatal, Child and Adolescent Mortality, State Government of Victoria, Melbourne. 2014. Available at https://www2.health.vic.gov.au/getfile/?sc_itemid=%7B67445EAC-0BB0-4525-9535-CCBF646BDADB%7D&title=Victoria's%20Mother's%20and%20Babies%3A%20Victoria's%20Maternal,%20Perinatal,%20Child%20and%20Adolescent%20Mortality%202010%2F2011%20. Accessed 17 Jan 2016.

[28] Davey MA, Sloan ML, Palma S, Riley M, King J (2013). Methodological processes in validating and analysing the quality of population-based data: a case study using the Victorian Perinatal Data Collection. HIM J.

[29] Dobbins TA, Sullivan EA, Roberts CL, Simpson JM (2012). Australian national birthweight percentiles by sex and gestational age, 1998-2007. Med J Aust.

[30] Pink B (2008). Information paper: an introduction to socio-economic indexes for areas (SEIFA), 2006.

[31] Puthussery S. Perinatal outcomes among migrant mothers in the United Kingdom: Is it a matter of biology, behaviour, policy, social determinants or access to health care? Best practice & research. Clinical Obstetrics & Gynaecology. 2015. 10.1016/j.bpobgyn.2015.09.003 26527304

[32] Ekeus C, Cnattingius S, Essen B, Hjern A (2011). Stillbirth among foreign-born women in Sweden. Eur J Public Health.

[33] Urquia ML, Qiao Y, Ray JG, Liu C, Hjern A (2015). Birth outcomes of foreign-born, native-born, and mixed couples in Sweden. Paediatric & Perinatal Epidemiology.

[34] Dahlen HG, Schmied V, Dennis CL, Thornton C (2013). Rates of obstetric intervention during birth and selected maternal and perinatal outcomes for low risk women born in Australia compared to those born overseas. BMC Pregnancy Childbirth.

[35] McMichael C, Manderson L (2004). Somali women and well-being: Social networks and social capital among immigrant women in Australia. Hum Organ.

[36] Abdelkerim AA, Grace M (2012). Challenges to employment in newly emerging African communities in Australia: a review of the literature. Aust Soc Work.

[37] Khashan AS, Everard C, McCowan LM, Dekker G, Moss-Morris R, Baker PN (2014). Second-trimester maternal distress increases the risk of small for gestational age. Psychol Med.

[38] Dejin-Karlsson E, Hanson BS, Ostergren PO, Lindgren A, Sjoberg NO, Marsal K (2000). Association of a lack of psychosocial resources and the risk of giving birth to small for gestational age infants: a stress hypothesis. BJOG.

[39] Zilko CEM, Rehkopf D, Abrams B (2010). Association of maternal gestational weight gain with short-and long-term maternal and child health outcomes. Am J Obstet Gynecol.

[40] Resnik R (2007). One size does not fit all. Am J Obstet Gynecol.

[41] Hutcheon JA, Zhang X, Platt RW, Cnattingius S, Kramer MS (2011). The case against customised birthweight standards. Paediatr Perinat Epidemiol.

[42] Hillier SL, Nugent RP, Eschenbach DA, Krohn MA, Gibbs RS, Martin DH (1995). Association between bacterial vaginosis and preterm delivery of a low-birth-weight infant. N Engl J Med.

[43] Lee AC, Katz J, Blencowe H, Cousens S, Kozuki N, Vogel JP (2013). National and regional estimates of term and preterm babies born small for gestational age in 138 low-income and middle-income countries in 2010. Lancet Global health.

[44] Richardus JH, Graafmans WC, Verloove-Vanhorick SP, Mackenbach JP (1998). The perinatal mortality rate as an indicator of quality of care in international comparisons. Med Care.

[45] Carolan M, Cassar L (2010). Antenatal care perceptions of pregnant African women attending maternity services in Melbourne, Australia. Midwifery.

[46] Correa-Velez I, Ryan J (2012). Developing a best practice model of refugee maternity care. Women Birth.

[47] Parker ED, Solberg LI, Foldes SS, Walker PF (2010). A surveillance source of tobacco use differences among immigrant populations. Nicotine Tob Res.

[48] Gibson-Helm ME, Teede HJ, Cheng IH, Block AA, Knight M, East CE (2015). Maternal health and pregnancy outcomes comparing migrant women born in humanitarian and nonhumanitarian source countries: a retrospective, observational study. Birth.

[49] Gagnon AJ, Zimbeck M, Zeitlin J (2010). Migration and perinatal health surveillance: an international Delphi survey. Eur J Obstet Gynecol Reprod Biol.

