# Supplementary material for: Perinatal health outcomes of East African immigrant populations in Victoria, Australia: a population based study
Source: BMC Pregnancy Childbirth. 2016 Apr 26;16:86. doi: 10.1186/s12884-016-0886-z (PMC4845379; doi:10.1186/s12884-016-0886-z)
Supplement: Additional file 1: Table S1. — Crude and adjusted odds ratios with (95 % CI) for selected perinatal health outcomes of East African immigrants and Australian-born women giving birth to singletons at ≥22 weeks of gestation in Victoria, Australia between 1999 and 2007. (DOC 68 kb) [file 12884_2016_886_MOESM1_ESM.doc]

Additional file 1: Table S1. Crude and adjusted odds ratios with (95%CI) for selected perinatal health outcomes of East African immigrants and Australian-born women giving birth to singletons at ≥22 weeks of gestation in Victoria, Australia between 1999 and 2007

| Outcomes | Australia  (N= 426,852) | Eritrea  (N=453) | Ethiopia  (N=1093) | Somalia  (N= 1,858) | Sudan  (N= 1,399) | Total East Africa  (N= 4,803) |
| --- | --- | --- | --- | --- | --- | --- |
| **Low birthweight (n and %)** | 21,599 (5.1) | 30 (6.2) | 56 (5.2) | 81 (4.4) | 89 (6.4) | 256 (5.3) |
| OR (95%CI) | 1.00 | 1.33 (0.92, 1.93) | 1.01 (0.77, 1.33) | 0.86 (0.68, 1.07) | **1.27 (1.03, 1.58)** | 1.06 (0.93, 1.20) |
| OR_adj_ (95%CI) | 1.00 | 1.46 (1.00, 2.12) | 0.92 (0.70, 1.22) | 0.90 (0.72, 1.13) | **1.37 (1.11, 1.71)** | 1.08 (0.96, 1.24) |
| **Very low birthweight (n and %)** | 8,246 (1.9) | 16 (3.5) | 35 (3.2) | 36 (1.9) | 39 (2.8) | 126 (2.6) |
| OR (95%CI) | 1.00 | **1.86 (1.13, 3.06)** | **1.68 (1.20, 2.35)** | 1.00 (0.72, 1.40) | **1.46 (1.06, 2.00)** | **1.37 (1.14, 1.63)** |
| OR_adj_ (95%CI) | 1.00 | **2.03 (1.22, 3.34)** | 1.52 (1.00, 2.15) | 1.05 (0.75, 1.47) | **1.55 (1.12, 2.13)** | **1.39 (1.17, 1.67)** |
| **Preterm birth (n and %)** | 25,997 (6.1) | 28 (5.8) | 69 (6.3) | 79(4.3) | 78 (5.6) | 254 (5.3) |
| OR (95%CI) | 1.00 | 1.01 (0.69, 1.49) | 1.04 (0.81, 1.33) | **0.68 (0.55, 0.86)** | 0.91(0.72, 1.14) | **0.85 (0.75, 0.97)** |
| OR_adj_ (95%CI) | 1.00 | 1.06 (0.72, 1.56) | 0.97 (0.76, 1.25) | **0.70 (0.56, 0.88)** | 0.94 (0.75, 1.18) | **0.87 (0.76, 0.98)** |
| **Very preterm birth (n and %)** | 4,777 (1.1) | 11 (2.4) | 27 (2.5) | 25 (1.4) | 28 (2.0) | 91 (1.9) |
| OR (95%CI) | 1.00 | **1.80 (1.01, 3.33)** | **2.24 (1.53, 3.28)** | 1.21 (0.81, 1.79) | **1.80 (1.24, 2.62)** | **1.71 (1.38, 2.10)** |
| OR_adj_ (95%CI) | 1.00 | **2.37 (1.30, 4.33)** | **1.99 (1.35, 2.95)** | 1.27 (0.85, 1.89) | **1.82 (1.25, 2.66)** | **1.71 (1.38, 2.11)** |
| **Apgar score <7 (n and %)** | 3,776 (0.94) | 6 (1.4) | 7(0.68) | 16 (0.90) | 18 (1.4) | 47 (1.0) |
| OR (95%CI) | 1.00 | 1.50 (0.67, 3.37) | 0.72 (0.34, 1.52) | 0.96 (0.59, 1.57) | 1.47 (0.92, 2.34) | 1.10 (0.83, 1.47) |
| OR_adj_ (95%CI) | 1.00 | 1.62 (0.72, 3.64) | 0.72 (0.35, 1.53) | 1.08 (0.66, 1.78) | 1.56 (0.97, 2.49) | 1.18 (0.89, 1.59) |
| **Macrosomia (n and %)** | 8,264 (1.9) | 6 (1.3) | 15 (1.4) | 37 (2) | 8 (0.6) | 66 (1.4) |
| OR (95%CI) | 1.00 | 0.68 (0.30, 1.52) | 0.70 (0.42, 1.17) | 1.03 (0.74, 1.43) | **0.29 (0.15, 0.58)** | **0.71 (0.55, 0.90)** |
| OR_adj_ (95%CI) | 1.00 | 0.67 (0.30, 1.51) | 0.70 (0.42, 1.16) | 0.94 (0.68, 1.30) | **0.26 (0.13, 0.53)** | **0.65 (0.52, 0.84)** |
| **SGA (n and %)** | 37,735 (8.8) | 54 (11.9) | 104 (9.5) | 222 (11.9) | 219 (15.5) | 599 (12.4) |
| OR (95%CI) | 1.00 | **1.40 (1.05, 1.85)** | 1.08 (0.89, 1.33) | **1.40 (1.22, 1.61)** | **1.91 (1.66, 2.21)** | **1.47 (1.35, 1.60)** |
| OR_adj_ (95%CI) | 1.00 | **1.53 (1.14, 2.04)** | 1.07 (0.88, 1.32) | **1.61 (1.39, 1.85)** | **2.19 (1.89, 2.54)** | **1.62 (1.48, 1.77)** |
| **Perinatal mortality (n /1000)** | 3,302 (7.7) | 11 (24.3) | 15 (13.7) | 28 (15.1) | 23 (16.4) | 77 (16) |
| OR (95%CI) | 1.00 | **3.19 (1.75, 5.81)** | **1.78 (1.07, 2.97)** | **1.96 (1.35, 2.85)** | **2.14 (1.42, 3.24)** | **2.09 (1.66, 2.62)** |
| OR_adj_ (95%CI) | 1.00 | **3.53 (1.93, 6.45)** | 1.58 (0.93, 2.69) | **2.09 (1.44, 3.05)** | **2.25 (1.48, 3.42)** | **2.13 (1.70, 2.70)** |

OR=crude odds ratio

OR_adj_= adjusted for maternal age, parity, marital status, SEIFA, year of birth and maternal medical complications
